# Global research publications on irrational use of antimicrobials: call for more research to contain antimicrobial resistance

**DOI:** 10.1186/s12992-021-00754-9

**Published:** 2021-08-24

**Authors:** Waleed M. Sweileh

**Affiliations:** Department of Physiology, Pharmacology/Toxicology, College of Medicine and Health Sciences, An-Najah National University, Nablus, Palestine

**Keywords:** Irrational use, Misuse, Antimicrobials, Antimicrobial resistance, Research

## Abstract

**Background:**

Irrational use of antimicrobials is highly prevalent. It is a major driving factor for antimicrobial resistance (AMR). Research on irrational antimicrobial use is important for developing policies and regulations to combat and contain AMR. The present study aims to provide an overview of research publications on the irrational use of antimicrobials at the national and global levels.

**Methods:**

Publications on irrational use of antimicrobials were extracted from Scopus using a wide range of relevant keywords for the study period from 1980 to 2020.

**Results:**

In total, 656 publications on irrational use of antimicrobials were found. The bulk of publications in this field were about irrational use in humans. A limited number of publications were found on the irrational use of antimicrobials in the context of veterinary and environment. The number of publications, contributing countries, and the mean number of authors per article increased with time, most notably in the last decade. Authors from 105 different countries participated in publishing the retrieved articles with 22 (21.0%) participated in 10 or more publications. The United States led with 140 (21.6%) articles followed distantly by China (*n* = 49, 7.5%), India (*n* = 45, 6.9%), and the United Kingdom (*n* = 45, 6.9%). Countries in the South-East Asian region (*n* = 69, 10.5%) and the African region (*n* = 42, 6.4%) made the least contribution. The list of most frequent author keywords included “antimicrobial stewardship” and “community pharmacies”. The research themes focused on the hospital-based rational use of antimicrobials and the self-medication practices with antimicrobials in the community. In total, 420 different journals participated in publishing the retrieved documents. The *Plos One* journal (17, 2.6%) ranked first. The retrieved articles received an average of 15.6 citations per article and an *h*-index of 52. The most frequent antimicrobials encountered in the retrieved literature were penicillin, cephalosporin, and fluoroquinolones while the most frequently encountered pathogens were *S. aureus* and *P. aeruginosa*.

**Conclusion:**

Research on the irrational use of antimicrobials is needed from all countries and regions to implement appropriate policies to contain the AMR. Research on irrational use of antimicrobials in the context of veterinary is needed.

## Introduction

Antimicrobial resistance (AMR) is a global public health challenge that threatens the ability of modern medicine to combat infectious diseases [1]. Several published reports indicated that AMR has reached an alarming stage [2]. The increasing level of AMR is expected to increase the rates of mortality and global economic burden [3]. The development of AMR threatens the achievement of sustainable development goals (SDGs), specifically SDG-03 about health and well-being [4]. That is why AMR has been declared as one of the global health threats to humanity [5]. In the European Union (EU), it was estimated that AMR is responsible for an estimated 33,000 deaths per year and costs the EU EUR 1.5 billion per year in healthcare costs and productivity losses [6].

The irrational or misuse of antimicrobials in humans or animals is a major driving factor for the development of AMR [7]. The World Health Organization (WHO) and the World Bank gave a broad definition of the rational use of medicines [8]. The definition focuses on the appropriate use of medicines based on the clinical needs of the patient or according to the scientific data and in a cost-effective way [9, 10]. Irrational use of medicines is present in all countries but mostly in developing countries due to the fragile or fragmented health system [11]. Major driving forces for irrational use of antibiotics include lack of adequate knowledge on the behalf of the patients or prescriber, easy access to antimicrobials without prescriptions, pharmaceutical promotion, parental pressure on prescribers, lack of rapid microbial testing, need for larger amounts of animal food, and poor communication among health professionals in the health system [12].

Self-medication with antibiotics is a common practice where patients self-diagnose and purchase antibiotics without prescription [13]. Self-medication is associated with the development of AMR [14]. The recent COVID-19 pandemic increased the inappropriate use of antibiotics, such as azithromycin, due to misinformation regarding the role of such antibiotics in treating COVID-19 infection [15]. The irrational use of antimicrobials could be minimized in hospital settings through the implementation of antimicrobial stewardship programs [16] where health professionals work together to give patients the appropriate antibiotics in the correct dose and for the correct duration based on rapid microbial testing. The misuse or overuse of antimicrobials in food-producing animals could be minimized by imposing regulations and restrictive policies on veterinary use of antimicrobials [17]. The AMR problems due to irrational or overuses of antimicrobials in food-producing animals is a serious problem that did not receive adequate attention. The emergence of plasmid-mediated resistance against colistin in pigs in China [18] was a warning signal about the misuse of antimicrobials in food-producing animals. The mobilized colistin resistance-1 gene (MCR-1), identified in 2014 in China, soon became a worldwide problem in human medicine [19]. The development and spread of AMR are expected to increase globally because of the increased number of populations accompanied by an increase in consumption of animal foods with potential environmental contamination with antibiotic wastes [20].

Assessment of national and global research publications on the irrational use of antimicrobials and its implications on national health policies is the first step to be undertaken in the fight against AMR. Low research volume might indicate poor national commitment to participate in the global fight against AMR, lack of government funding for scholars, and lack of research interest or expertise to investigate irrational behaviors and practices leading to AMR. Research publications on irrational use of antimicrobials are important for (1) building nationally-tailored policies to combat AMR, (2) increasing public awareness and information by disseminating results and recommendations through national media, and (3) developing evidence-based treatment guidelines since the AMR profile of various pathogens change with irrational practices.

Assessment of the growth and developmental pattern of research publications on any topic could be carried out using bibliometric analysis, defined as the application of mathematics and statistics on related publications [21]. Scanning academic databases such as Pubmed, Scopus, and Web of Science showed that no bibliometric studies on the irrational/misuse/overuse of antimicrobial agents have been published. Therefore, the present study was undertaken to assess national and global research activity on the irrational use of antimicrobials using bibliometric tools. The ultimate goal of the study was to encourage academics, medical professionals, and health policymakers to get engaged in research that helps minimize or stop the acceleration of the AMR problem.

## Methodology

### Type of the study

This was a descriptive cross-sectional study of publications on irrational use of antimicrobials using bibliometric tools.

### Database used

The largest database to retrieve the maximum number of related documents on any subject is Google Scholar. However, Google Scholar lacks metric analysis. Another large database with functions for metric analysis is the Scopus database with approximately 24,000 indexed journals in all scientific fields. Therefore, Scopus was used in the present study despite it has a lesser number of indexed journals than Google Scholar [22, 23].

### Search strategy

The absence of systematic reviews or scoping reviews or bibliometric analysis on irrational use/misuse of antimicrobials made the development of a valid and comprehensive search strategy difficult. Therefore, the author developed the search strategy based on common terms used in the published literature about the irrational use/ misuse of antimicrobials.

The Scopus database has basic and advanced search options. A large number of terms could be used in the advanced search function. In the advanced search option, both the quotation marks and asterisk are used to facilitate the retrieval of an accurate and large number of documents. The advanced search option allows the use of Boolean operators such as OR, AND, NOT, LIMIT, EXCLUDE, and many others.

In the current study, terms related to antimicrobials/antibiotics included: “antibiotic*” or “antimicrobial*” or penicillin or cephalosporin or fluoroquinolone or macrolide or carbapenem or vancomycin or aminoglycoside or antibacterial or antiviral or antimalarial or anti-TB or antimycobacterial or tetracyclin* or quinolones or anti-retroviral or colistin or antiparasitic or antifungal. Termsrelated to irrational use or misuse included: “without prescription” or “self-prescription” or “self-prescribing” or “irrational use” or “inappropriate use” or “inappropriate prescri*” or “antibiotic overuse” or “overuse of antibiotics” or “non-adherence to antibiotic*” or “patient misinformation” or “misuse of antib*” or “excessive use” or “self-medication with antibiotic*” or “self-therapy” or “inappropriate use” or “inappropriate utilization” or “irrational use” or “over prescribing” or “antibiotic polypharmacy” or “non-adherence to antibiotic*” or “unjustifi* use” or “unnecessary use” or “poor knowledge” or “inadequate information” or “lack of awareness” or “no regulation” or “absence of regulation” or “no otc guideline*” or “responsible antibiotic use” or “irrational antibiotic use” or “inappropriate antibiotic use” or “non-prescription sale” or “non-prescription dispensing” or “antibiotic abuse” or “*rational use of antibiotic*” or “*rational use of antimicrobial*” or “knowledge about antibiotic*” or “knowledge and attitude about antibiotic*” or “under prescribing of antibiotic*” or “under use of antibiotic*” or misdiagnosis or “incorrect choice” or “poor prescribing practice*” or “incorrect dosage” or “incorrect dosing” or “irrational dispensing” or “antibiotic use and misuse” or “use and misuse of antibiotic*” or “antibiotic misuse” or “misuse of antibiotic*” or “casual use of antibiotic*”. The overall search strategy consisted of terms related to antimicrobials combined with terms related to irrational use.

### Validation of the search strategy

No search strategy is 100% perfect. However, the search strategy must be valid in terms of having a minimum number of false-positive and false-negative results. Therefore, the search strategy was tested for the absence of false-positive results by reviewing a random sample of 100 documents. The review was carried out by independent reviewers in the field of medicine/pharmacy. The search strategy was fine-tuned based on the feedback from the reviewers. The search strategy was finalized when the reviewers found no false-positive results in the random sample of selected documents. The search strategy was not tested for the absence of false negatives because of the lack of previous publications in this field to compare the results. However, a quick test was made by investigating the number of documents published in the *Plos One* journal on irrational use/misuse and compared with that obtained by the search strategy. The search strategy returned approximately 17 documents while the manual search in the *Plos One* journal returned 19. This means that the search strategy was capable of retrieving approximately 90% of the published literature on the irrational use/misuse of antimicrobials.

### Inclusion and exclusion criteria

In the present study, research and review articles in addition to conference papers were included in the analysis. Other types of documents such as notes, editorials, letters, books, and book chapters were excluded. Furthermore, only documents published between 1980 and 2020 were included. This period represents the times before and after the AMR crisis. The results of the search strategy were not limited to any type of language.

### Data management

The retrieved data were exported from Scopus to Microsoft Excel for bibliometric analysis and mapping which included: (1) leading countries; (2) the number of documents contributed by each WHO region (the region of Americas; the European region, the African region; the Eastern Mediterranean region, the South-East Asian region, and the Western Pacific region); (3) leading authors; (4) number of citations; (4) the most frequent author keywords; (5) mapping of research collaboration; and (6) mapping of most frequent terms in the titles/abstracts. The mapping was carried out using VOSviewer [24] while linear graphs were made by the Statistical Program for Social Sciences.

## Results

The search strategy returned 656 documents: 575 (87.6%) research articles, 72 (11.0%) review articles, and 9 (1.4%) conference papers. Of the retrieved documents, only 10 discussed the irrational use/misuse of antimicrobials in the context of veterinary. Therefore, the bulk of the retrieved documents discussed irrational use/misuse of antimicrobials in humans.

### Growth trajectory

**Fig. 1:**
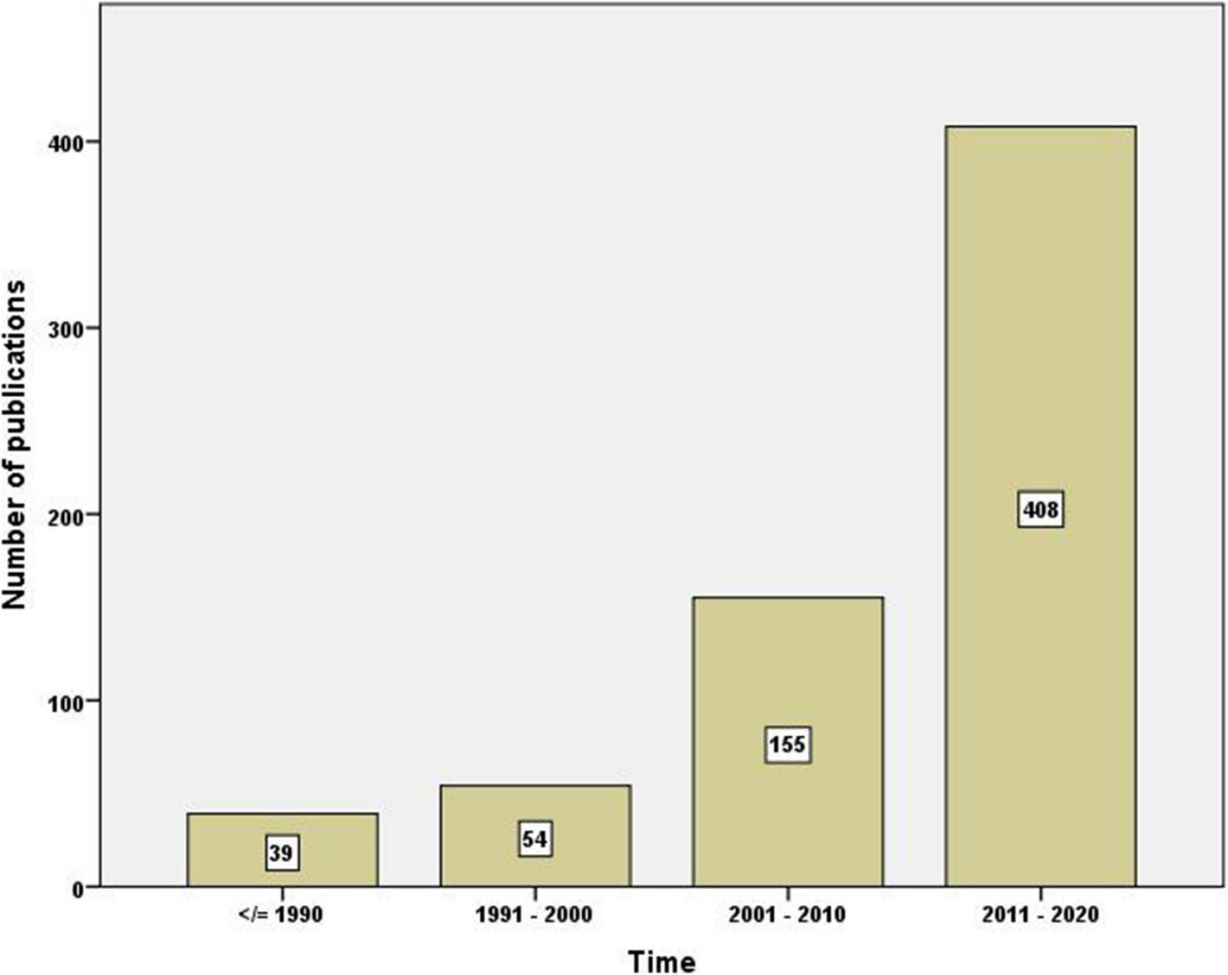
Number of research publications on irrational use of antimicrobials per decade

**Fig. 2:**
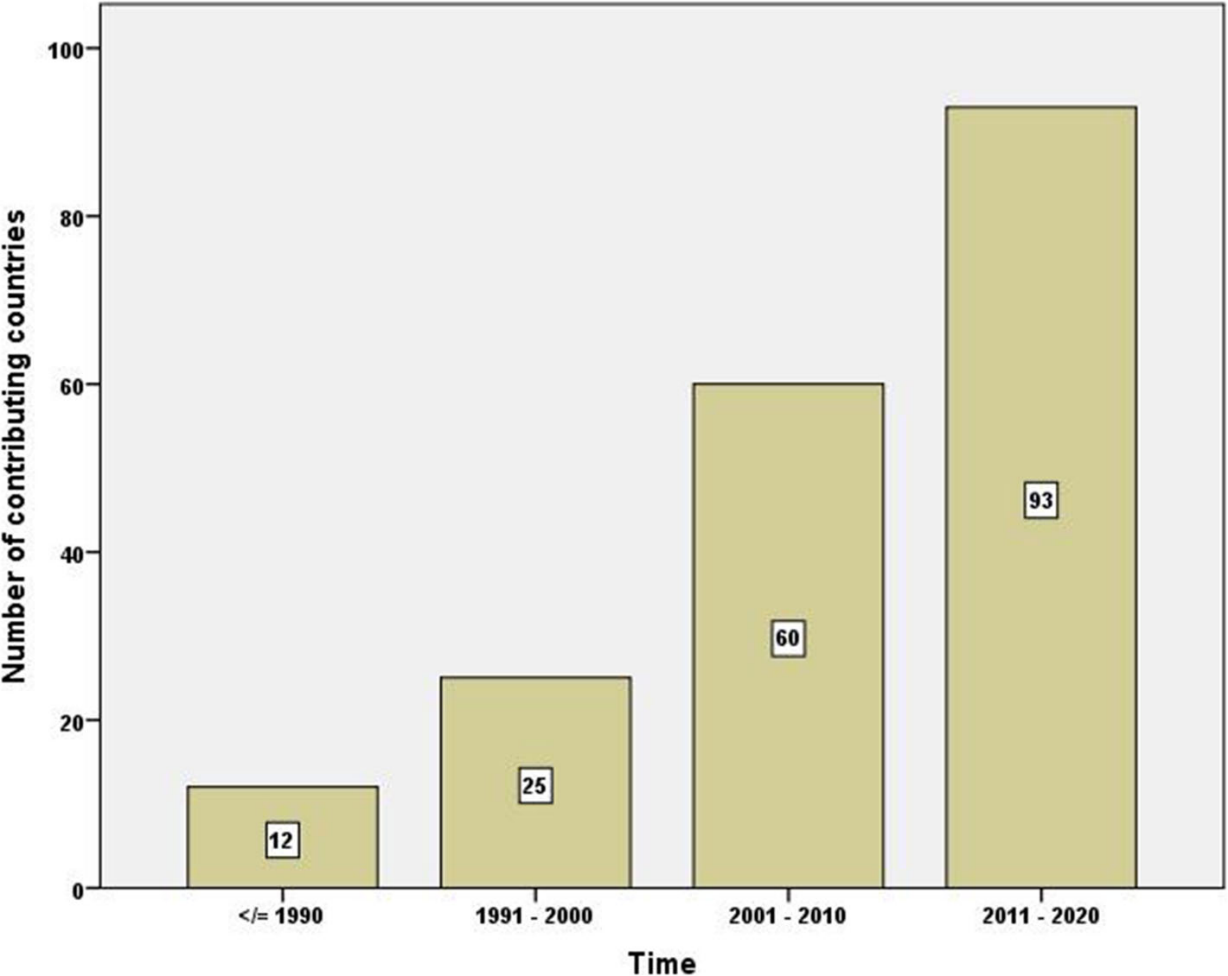
Number of countries contributing to research publications on irrational use of antimicrobials per decade

**Fig. 3:**
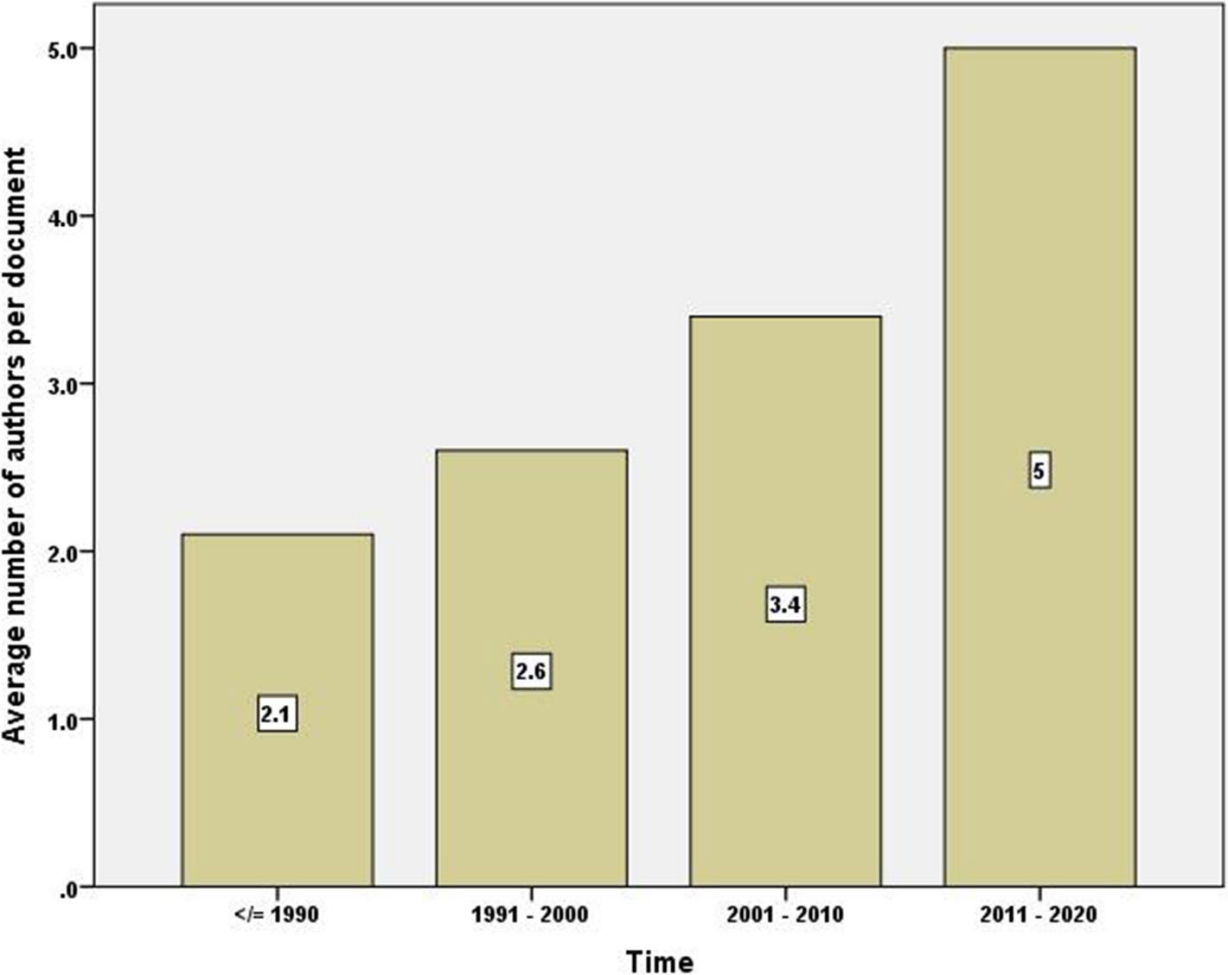
Average number of authors per article for research publications on irrational use of antimicrobials per decade

Figure 1 is a bar chart showing the number of publications per decade. The figure shows a 10-fold increase in the number of publications between the first and last decade of the study. A similar pattern of the increase was seen in the number of countries participating in publishing documents on irrational use with time (Fig. 2). The average number of authors per document also increased with time (Fig. 3). The average number of authors per document was 5.0 for articles published in the last decade compared to 2.1 authors for articles published in the first decade of the study.

### Leading countries/world regions

Overall, authors from 105 different countries participated in publishing the retrieved documents. Of the 105 countries, 22 (21.0%) had research productivity of 10 or more publications while 23 (21.0%) had research productivity of one article for each country. Table 1 shows the list of countries with a minimum contribution of 10 publications. The list included countries from all world regions. The United States (US) led with 140 (21.6%) documents followed distantly by China (*n* = 49, 7.5%), India (*n* = 45, 6.9%), and the United Kingdom (UK) (*n* = 45, 6.9%).

**Table 1:** List of countries with a minimum contribution of 10 papers on irrational use of antimicrobials

Country	Number of publications	% (***N*** = 656)
United States	140	21.3
China	49	7.5
India	45	6.9
United Kingdom	45	6.9
Netherlands	32	4.9
Australia	28	4.3
Spain	28	4.3
Saudi Arabia	27	4.1
Sweden	22	3.4
Canada	20	3.0
France	19	2.9
Pakistan	17	2.6
Germany	16	2.4
Italy	16	2.4
Switzerland	14	2.1
Turkey	14	2.1
Belgium	13	2.0
Greece	12	1.8
Malaysia	12	1.8
Nigeria	11	1.7
Brazil	10	1.5
South Africa	10	1.5

**Fig. 4:**
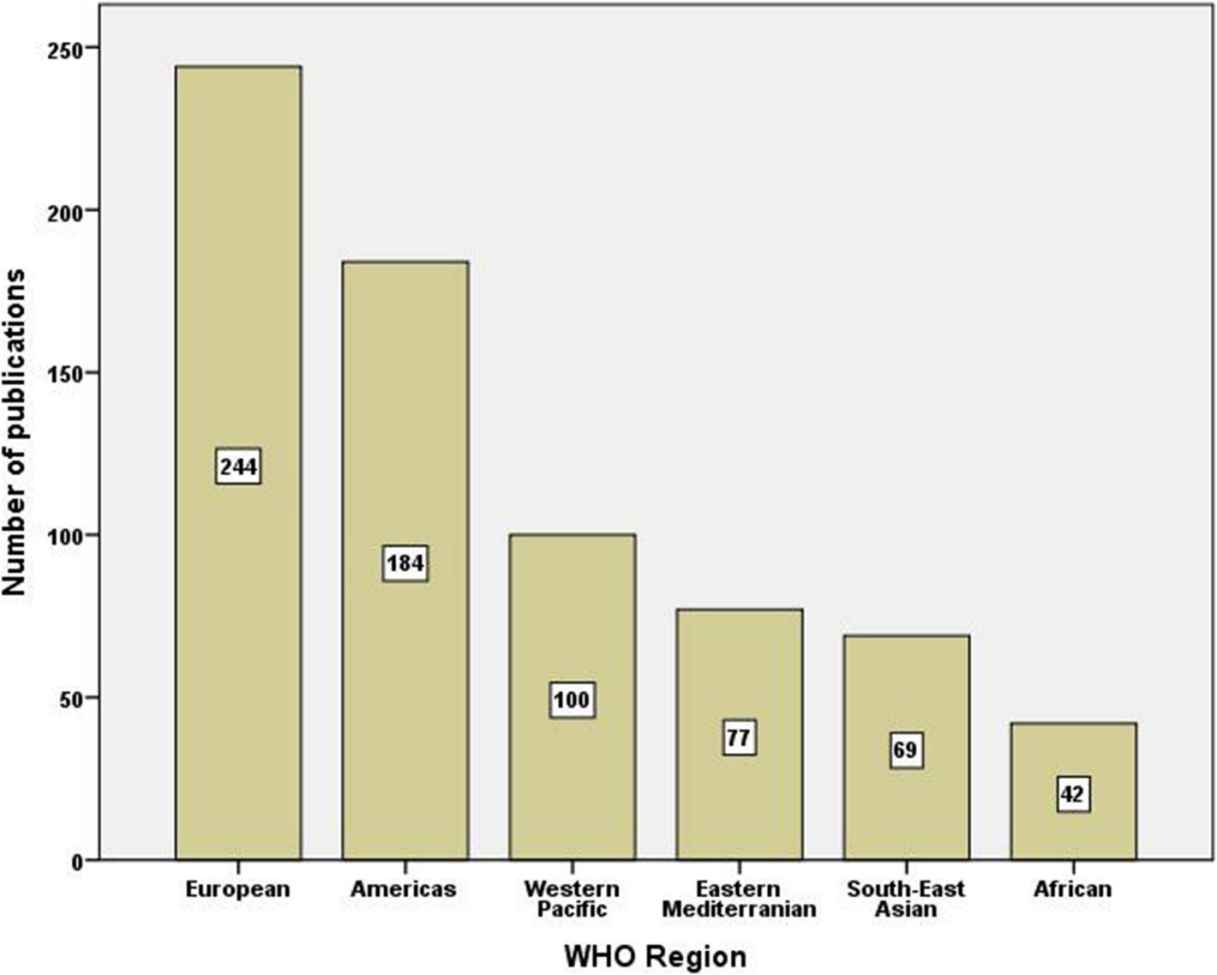
Number of publications on irrational use of antimicrobials from different world regions

Geographic distribution of the retrieved documents showed that the WHO Region of the Americas (North and South America) had the highest share (*n* = 184, 28%) followed by the European region (*n* = 244, 37.2%), the Western Pacific region (*n* = 100, 15.2%), the Eastern Mediterranean region (*n* = 77, 11.7%), the South-East Asian region (*n* = 69, 10.5%), and the African region (*n* = 42, 6.4%) (Fig. 4).

### Most frequent author keywords

The top 30 frequent author keywords were those mostly present in the search strategy in addition to other related author keywords such as antimicrobial stewardship, guidelines, hospital, children, public health, community pharmacy, and pharmacy practice (Table 2).

**Table 2:** Top 30 author keywords in the retrieved papers on irrational use of antimicrobials

Author keyword	Number of occurrences	Author keyword	Number of occurrences
antibiotics	156	community pharmacy	11
self-medication	65	Saudi Arabia	10
antibiotic resistance	39	antimicrobial stewardship	9
antibiotic	36	misuse	9
resistance	27	rational use	9
antimicrobial resistance	26	antibiotic overuse	8
antibiotic use	20	China	8
anti-bacterial agents	15	pharmacy	8
knowledge	15	rational use of antibiotics	8
children	14	attitude	7
drug resistance	14	guidelines	7
antibiotic stewardship	13	hospital	7
prescription	13	infection	7
bacterial resistance	12	practice	7
antibiotic misuse	11	public health	7

### Research themes

**Fig. 5:**
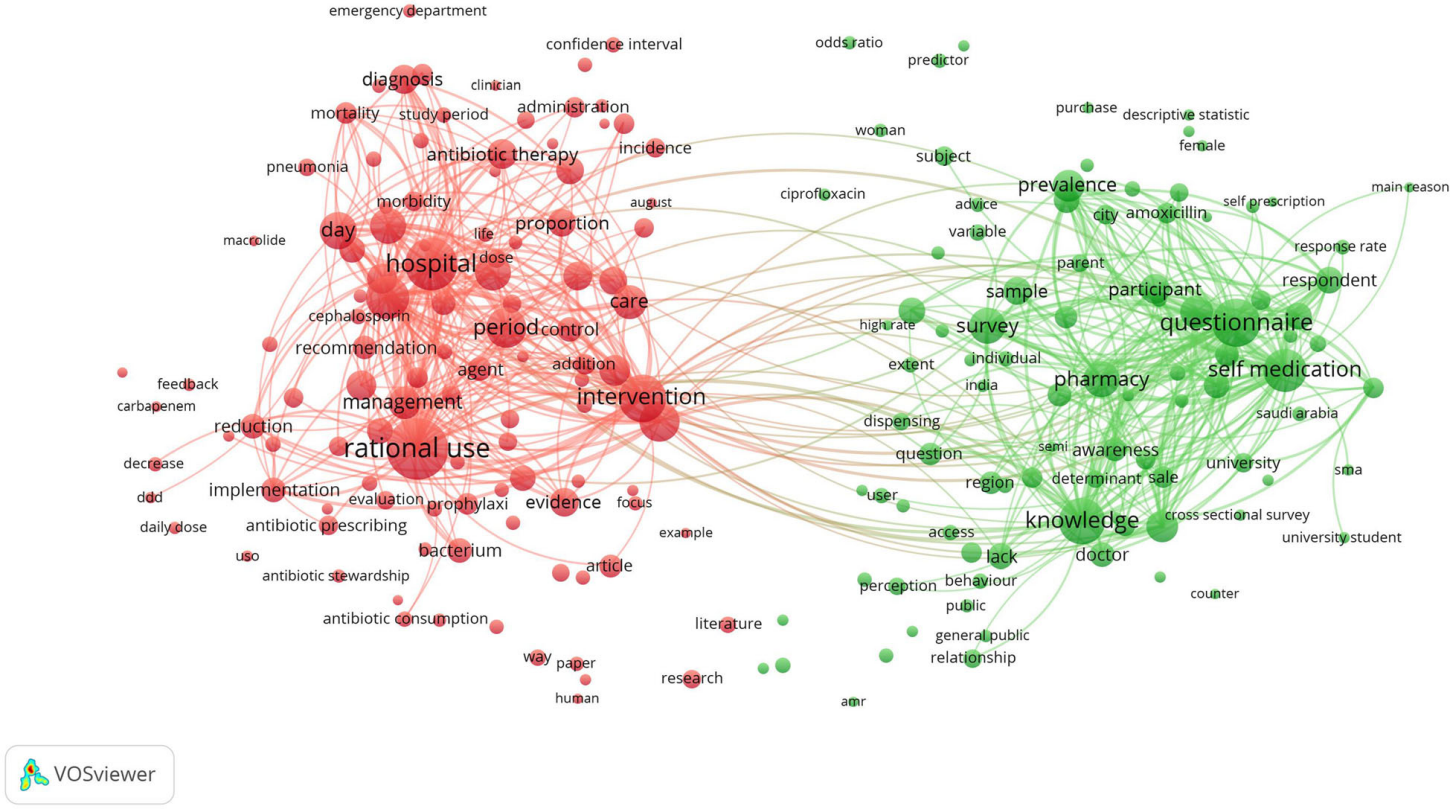
Network visualization map of terms in the titles and abstracts of the retrieved publications. The threshold of inclusion was 10 occurrences

Mapping terms in the titles and abstracts with a minimum frequency of 10 occurrences showed two large clusters representing two major research themes: hospital-based studies and intervention for rational use of antibiotics and community-based studies on self-medication with antibiotics (Fig. 5).

### Most commonly encountered anti-infective agents

**Fig. 6:**
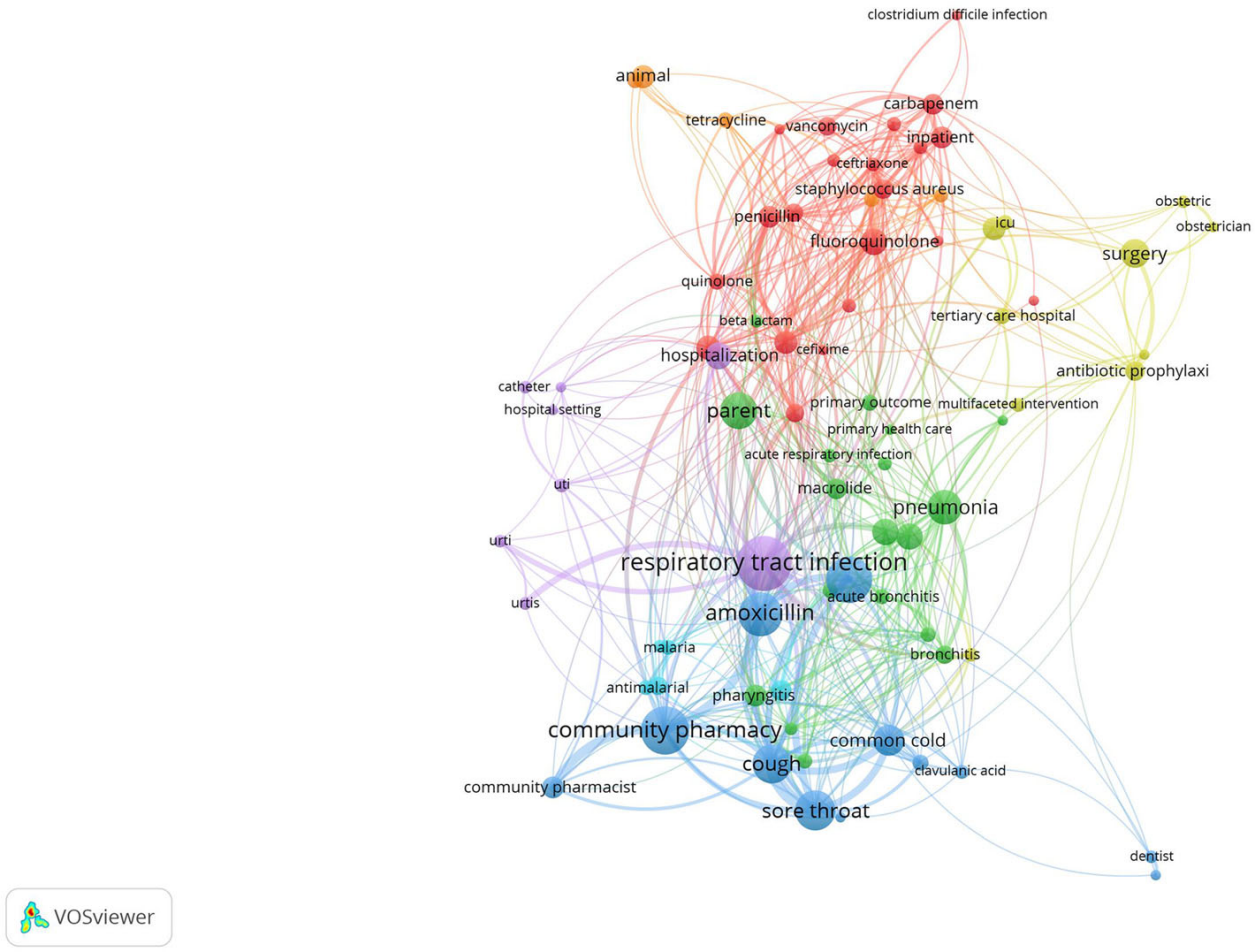
Network visualization map of antimicrobials and pathogens with minimum occurrence of 3 in the titles/abstracts of the retrieved publications

Analysis of anti-infective agents associated with irrational and misuse of antimicrobial agents indicated that amoxicillin was most frequently encountered followed by fluoroquinolones, cephalosporin, and vancomycin. Infections most commonly associated with irrational use were mainly respiratory tract infections (*n* = 66) and to a lesser extent urinary tract infections (*n* = 27), and diarrhea (*n* = 26). Of the respiratory tract infections, cough, pneumonia, and common cold were most frequent. The most common pathogens encountered included *S. aureus* and *P. aeruginosa* (Fig. 6).

### Leading journals

The retrieved documents were published in 420 different journals. Sixteen (3.8%) journals published five or more documents while 311 (74.0%) journals participated in one article each. Table 3 shows the list of journals with a minimum of five publications. The *Plos One* journal (17, 2.6%) ranked first. The list included nine journals in the field of infectious diseases, two in the field of public health, and 4 in the field of pharmacy.

**Table 3:** Journals with a minimum contribution of five papers on irrational use of antimicrobials

Journal	Number of publications	% (***N*** = 656)
*Plos One*	17	2.6
*Journal Of Antimicrobial Chemotherapy*	15	2.3
*BMC Public Health*	9	1.4
*Infection Control And Hospital Epidemiology*	8	1.2
*Clinical Infectious Diseases*	7	1.1
*Expert Review Of Anti Infective Therapy*	7	1.1
*Antimicrobial Resistance And Infection Control*	6	0.9
*BMC Infectious Diseases*	6	0.9
*International Journal Of Antimicrobial Agents*	6	0.9
*International Journal Of Environmental Research And Public Health*	6	0.9
*Antibiotiki I Khimioterapiya*	5	0.8
*Infection And Drug Resistance*	5	0.8
*International Journal Of Clinical Pharmacy*	5	0.8
*Latin American Journal Of Pharmacy*	5	0.8
*Pharmaceutical Care And Research*	5	0.8
*Pharmaceutical Care Espana*	5	0.8

### Citation analysis

Citation analysis indicated that the retrieved papers received 10,240 citations, an average of 15.6 per document, and an h-index of 52. The range of citations was from 0 to 351. Of the retrieved documents, 219 received one citation or less while 234 documents received 10 or more citations.

## Discussion

The present study aimed to give an overview of global research publications on irrational use and misuse of antimicrobial agents in humans. The findings of the present study showed an overall increase in the number of publications, contributing countries, and the average number of authors per document with time. However, the number of countries with sizable contributions was limited, the average number of citations per document was relatively low, and the number of journals with prominent contributions was also limited. Irrational use of antimicrobial agents was mainly associated with amoxicillin, cephalosporins, and fluoroquinolones in the treatment of respiratory tract infections, specifically viral infections. At the community level, the bulk of literature focused on self-medication with antibiotics and the role of community pharmacies, and the absence of regulation in this field.

In response to increasing reports of irrational use of antimicrobials and the emergence of the AMR problem, the World Health Assembly Resolution urged member states to develop and adopt strategies that promote the rational use of antimicrobial agents to minimize the spread of resistant pathogens [25] and that is why the WHO developed the WHO global strategy for containment of AMR in 2001. These reports attracted the attention of many countries, researchers, academics, and policymakers. The present study showed that the rise in the number of publications on irrational and misuse of antimicrobial agents and its implications on AMR were noticed between 1998 and 2002. In 2014, the WHO warned of a post-antibiotic era where minor infections become potentially fatal [26]. In 2015, the Global Action Plan (GAP) on AMR was developed and endorsed by the Food and Agriculture Organization of the United Nations (FAO) and the World Organization for Animal Health (OIE). Countries committed to the implementation of GAP were asked to develop national plans to contain AMR in a one-health approach [27, 28]. In the last 5 years of the study period (2016–2020), approximately 37% of the retrieved documents were published. The increased number of contributing countries to the literature on irrational and misuse of antimicrobial agents has increased with time. However, approximately half of the countries in the world did not participate suggesting poor monitoring and regulations regarding antimicrobial resistance. These countries are located in WHO regions with limited human and financial resources such as the African region, the Eastern Mediterranean region, and the South-East Asian region. Between 2000 and 2010, the human consumption of antibiotics increased by 36% per capita, and a sharp increase was noted in low- and middle-income countries [29]. The sharp increase in consumption is indicative of poor research, poor awareness, inadequate policies and regulations, lack of strong health systems, and lack of adequate health services. A study in Tanzania investigated antibiotic purchases from drug outlets and found that 135 (88.8%) of antibiotic purchases were irrational and poor knowledge about the use of antibiotics was significantly associated with the irrational use of antibiotics [30]. The findings in the present study that the average number of authors per document increased significantly with time is attributed to the increasing number of scholars who showed interest in the field of AMR and the multidisciplinary nature of research on irrational use of antimicrobial agents. The AMR is considered of interest to researchers in public health, microbiology, pharmacology, infections, economy, and even politics.

Mapping author keywords showed that antimicrobial stewardship (AMS)/antibiotic stewardship (ABS) was among the top frequent author keywords. Antimicrobial stewardship programs are designed to minimize the irrational use of antimicrobials in hospital settings [31, 32]. The AMS programs minimize the irrational use of antibiotics and therefore proven cost-effective, safer to the patient, and effective in minimizing the development of AMS [33, 34]. Another frequent author keyword was “community pharmacy”. Self-medication and sale of antimicrobials without prescription often takes place in community pharmacies specifically in low- and middle-income countries. A recent study in Egypt assessed the dispensing patterns in Egyptian community pharmacies found that amoxicillin was dispensed to 98% of the patients despite that the patients had simulated viral respiratory infections [35]. A comprehensive systematic review on self-medication with antibiotics included 140 studies of all ages and diverse geographical locations [36]. The majority of the studies included in the analysis were from Brazil (12; 9%), followed by India (9; 6%), Pakistan (9; 6%), and Nigeria (8, 6%). The study found that the most widely self-medicated drug classes were antibiotics, followed by NSAIDs, and cough and cold medicines. A second systematic review of studies on self-medication and self-prescription of antibiotics in the Middle East found 22 studies [37]. The study found that penicillin was the most commonly used and the main complaint was upper respiratory tract problems. These reported results are in agreement with the findings of the present study regarding respiratory tract problems and amoxicillin as the chief complaint and the main antibiotic associated with irrational use/self-medication. The use of amoxicillin in self-medication practices was reported in several published studies from different geographic locations [38, 39]. The non-prescription use of antibiotics is not limited to low- and middle-income countries. Such practices have been reported in high-income countries but under-studied [40, 41]. The published studies on self-medication with various types of antimicrobial agents appeared as one research theme upon mapping the most frequent terms in the titles/abstracts of the retrieved documents. Self-medication and self-prescription with antibiotics are facilitated by a lack of monitoring and the absence of strict regulations regarding the sale or dispensing of antibiotics [42]. A systematic review study on global access to antibiotics without prescription in community pharmacies returned 38 studies from 24 countries [43]. The study concluded that the overall pooled proportion of the non-prescription supply of antibiotics following a patient request was 78% despite that all included countries, except for one, classified antibiotics as prescription-only medicines. The study also concluded that fluoroquinolones and penicillin respectively were the most commonly supplied antibiotic classes for complaints such as urinary tract and respiratory tract problems.

The present study showed that no single journal showed dominance in publishing documents on the irrational use of antibiotics. However, journals in the field of infection and AMR were most common. This was expected given that irrational use of antimicrobials will ultimately affect the efficacy of antimicrobials in treating and controlling infections. Research on irrational use, misuse, and self-medication with antibiotics is usually of interest to a wide range of health-related journals making the number of journals involved in publishing the retrieved documents high. This might also explain the findings of the average number of citations per document. Despite the global health impact and the high prevalence of antibiotic misuse [44, 45], the results showed a relatively low number of publications and citations suggestive of an inadequate number of readers and scholars interested in the subject. One reason behind this is the idea that more interventional studies are needed to tackle the AMR rather than theoretical descriptive studies on drivers of the problem of AMR. Interventional studies should include monitoring community pharmacies for the sale of antibiotics without prescription, implementing programs to increase public knowledge and awareness of the consequences of irrational and misuse of antibiotics, and the implementation of antimicrobial stewardship in hospitals and clinical settings to minimize cost and development of AMR.

### Recommendations and policy implications of the current study

The current study has several implications on national and international health policies regarding irrational use/misuse of antimicrobials.

1. Research activity on the topic enables policymakers and public health experts to quantify the problem and assess its impact on AMR.
2. One important point to strengthen research on the irrational use/misuse of antimicrobials is to establish national pharmacoepidemiology to investigate antimicrobial consumption and draw plans on how to minimize it.
3. The pharmaceutical industry should be fully involved in the fight against irrational/misuse/overuse of antimicrobials by monitoring and restricting the sale of antimicrobials to community pharmacies.
4. The finding that a limited number of publications were present in different parts of the world does not mean that the problem of irrational use of antimicrobials is absent. Lack of research publications mostly suggests a lack or absence of scholars interested in the field. The opposite is true. Countries with high research activity are not necessarily suffering from a widespread problem of irrational use. Investment of high-income countries in research collaboration in this field will reflect positively on the national health of developed countries because AMR knows no boundaries.
5. Research on irrational/misuse/overuse of antimicrobials is of importance to scholars in the field of medicine, public health, veterinary, nutrition, and the environment. Therefore, editors of journals in these fields should endorse publications in this field and publish thematic issues in this field.
6. Research on irrational use/misuse/overuse of antimicrobials in veterinary and the environment/ecosystem must be encouraged as an implementation of the “one health” approach.
7. Research on various policies and regulations to limit irrational use of antimicrobials need to be carried out and compared to encourage low- and middle-income countries to adopt similar regulations and policies.
8. Research on easy access and OTC dispensing of antimicrobials need to be investigated and linked to AMR. This is important to increase awareness of healthcare providers and the public about the potential driving force of the AMR.

### Limitations

The present study has a few limitations that are typical of any bibliometric study. Both the search query and the use of Scopus are always the limitation of bibliometric studies. Search queries might include false-positive or false-negative results but remain insignificant. The use of Scopus leads to an underestimation of research productivity from countries in world regions with un-indexed journals.

## Conclusions

The last decade has witnessed a significant increase in the number of publications, the number of contributing countries, and the number of authors per document. However, the present study showed that the volume of research publications on irrational use and misuse of antimicrobial agents is low relative to the prevalence and impact of irrational use of antimicrobial agents on global health. The bulk of research on the irrational use of antimicrobials originated from a limited number of countries and the bulk of research publications focused on misuse and self-medication with antibiotics in the context of community pharmacy practice. The remaining bulk of research publications focused on hospital settings and the role of antimicrobial stewardship. Information and research data on irrational use and misuse of antimicrobial agents are needed from all countries and world regions. Interventional studies need to be tailored based on the most common irrational practices. Irrational use of antimicrobials in the context of veterinary, agriculture, and environment are highly needed and should be encouraged,

## Data Availability

All data present in this article can be retrieved from Scopus using keywords listed in the methodology.

## Abbreviations

AMR: Antimicrobial resistance
WHO: World Health Organization
